# Salt tolerance enhancement Of wheat (*Triticum Asativium L*) genotypes by selected plant growth promoting bacteria

**DOI:** 10.3934/microbiol.2020016

**Published:** 2020-08-18

**Authors:** Alaa Fathalla, Amal Abd el-mageed

**Affiliations:** Department of agric. Botany, Faculty of agriculture, Suez canal university, Ismailia, Egypt

**Keywords:** genotypes, wheat, *Pseudomonas*, *Bacillus*, salinity

## Abstract

The study was conducted at green house and laboratories of Agriculture Botany Department, Faculty of Agriculture, Suez Canal University, Ismailia governorate, Egypt during 2018/2019 to test rhizosphere growth promoting bacteria as known strategy to increase salinity tolerance of six genotypes of wheat namely; Line 404, Line 356, Line 420, Line432, Sakha 93 and Line 380 were grown under 3000 ppm and 5000 ppm of salinity. Four bacterial strains were used namely; *Pseudomonas fluorescens* NBRC 14160, *Serratia liquefaciens* ATCC 27592, *Bacillus subtilis* SBMP4 and *Bacillus megaterium* NBRC 15308. All the strains could be able to tolerate salinity levels up to 3% NaCl and produced indole acetic acid (IAA). The both strains *Pseudomonas fluorescens* NBRC 14160 and *Bacillus megaterium* NBRC 15308 were grow on NA media supplemented with 6% NaCl, and showed 1-aminocyclopropane-1-carboxylate (ACC) deaminase activity and *Pseudomonas fluorescens* NBRC 14160 strain also fixed nitrogen. PCR results confirmed the previous results for both strains. *Pseudomonas fluorescens* NBRC 14160 and *Bacillus megaterium* NBRC 15308 were selected to study their reflection *in vivo* on wheat plants growth at different levels of salinity. The selected strains were able to improve plants growth under salinity stress conditions when compared with non-inoculated plants for all wheat genotypes especially sakha93 showed the highest mean values over rest genotypes under saline and non-saline conditions. Results of genetic parameters for studied traits showed that values of PCV were higher than GCV values for most studied traits. Germination percentage, shoot length and potassium content had high values of heritability and genetic advance, so these traits might use in selection of plant breeding programs for salinity tolerance.

## 1. Introduction

Wheat crop (*Triticum asativium L*.) is the most important nutrient cereal crop in the world [1]. Several types of abiotic stress causes reduction in production of many agricultural crops over the world [2],[3]. Salinity is mainly restricted problem to wheat grow; 20% of cultivated area and 50% of irrigated area are suffer from salinization process [4]. Plants grown under saline conditions are affected in many ways such as reduced water potential in root zone causing water deficit, phytotoxicity of ions such as Na+ and Cl–, nutrient imbalance depressing uptake and transport of nutrients and oxidative stress [5],[6]. The competition between nutrient elements like Na+ and K+ for binding sites causing physiological problems essential for cellular functions [7]. All of these effects caused reduction in net yield of wheat and destruction of soil properties.

Several traditional methods are used to avoid these problems like using tolerant genotypes and some agriculture practice to reduce the negative effect of salinity on wheat plants. In recent study was used number of useful rhizosphere microbes to increase the ability of salinity tolerance. Plant growth promoting rhizobacteria (PGPR) is beneficial plant-associated bacteria which enhance nutrition and help to cope biotic and abiotic stress [8]–[11]. The beneficial traits of plant growth promoting bacteria that alleviate salinity stress by enhancement plant growth via various mechanisms like nitrogen fixation, indole acetic acid (IAA) production, 1-aminocyclopropane-1-carboxylate (ACC) deaminase production, solubilization of phosphates and siderophores production [12]–[14].

PGPR inoculation was reported for its role in reducing the effects of salinity stress in various crops wheat, onion, chickpea, wheat, maize, tomato and groundnut etc. [15],[16]. Some of these PGPR belong to genera *Azospirillum*, *Azotobacter*, *Bacillus*, *Burkholderia*, *Erwinia*, *Enterobacter*, *Flavobacterium*, *Micrococcus*, *Pseudomonas* and *Serratia* etc. have been shown to enhance tolerance of salt in several crops [17],[18].

The main purpose for this research is using a biological option to ameliorate wheat under two different salinity levels in natural soil by pot experiments. Therefore, the present research was designed to study plant growth-promoting traits including Nitrogen fixation, production of indole acetic acid (IAA), swimming and swarming motility and 1-aminocyclopropane-1-carboxylate (ACC) deaminase. Furthermore, under different treatments growth, physiological and genetic parameters of six wheat genotypes were measured.

## 2. Material and methods

### 2.1. Salt tolerance assay

For observing salt tolerance strains, bacteria were grown on nutrient agar (NA) amended with different concentrations of NaCl (1–10%, w/v) and were incubated for 7 days at 28 °C [19].

### 2.2. Ammonia production and nitrogen fixation

The bacterial colonies were inoculated on N-free malate (NFB) solid medium at 28°C for 7 days as described by [20]. The media content were adjusted to pH 7 before autoclaved (121 °C for 20 minutes). Nitrogen fixation was Further confirmed by PCR amplification of partial *nifH*. For this purpose, a *nifH* gene was amplified by using two universal primers., 19F (5′-GCIWTYTAYGGIAAR GGIGG-3′) and 407R (5′-AAICCRCCRCAIACIACRTC-3′) which was described by [21].

### 2.3. 1-aminocyclopropane-1-carboxylate (ACC) deaminase activity

All strains were screened for their ability to use ACC as the sole nitrogen source in (DF) minimal salt medium according to the method of [22]. ACC deaminase production was further confirmed by PCR amplification by using the primers AccF 5′-ATGAATCTGAATCGTTTTGAAC-3′ and 5′-TCAGCCGTTGCGGAACAG-3′ were used to amplify *acdS* gene which was described by [23].

### 2.4. Determination of IAA produced by bacterial strains

For the indole acetic acid (IAA) produced by bacterial strains, it was determined by [24].

### 2.5. Swimming and swarming motility assay

Swimming and swarming motility was carried out using the method by [25]. The motility for Swimming and swarming were calculated after 48h by measuring the swarm and swim diameter and expressed in mm.

### 2.6. Plant experiment

**Figure 1.**
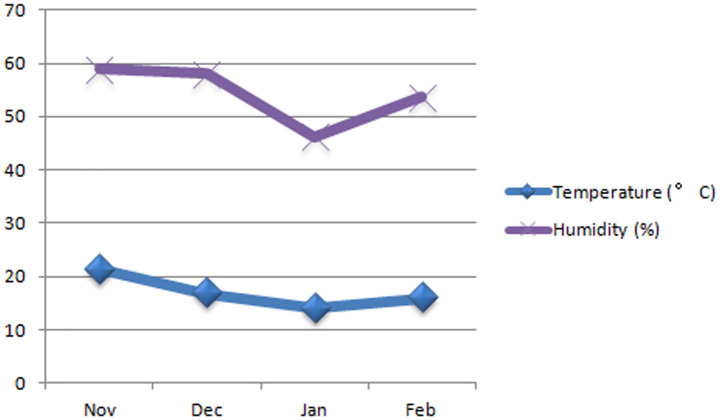
Average temperature range and relative humidity range during experimental planting period.

The experiments were carried out at green house and laboratories of Agriculture Botany department, Faculty of Agriculture, Suez Canal University, Ismailia governorate, Egypt during 2018/2019. Six wheat (*Triticum aestivum* L.) genotypes namely; Line404 (V1), Line356 (V2), Line420 (V3), Line432 (V4), Sakha 93 (V5) and Line 380 (V6) obtained from ICARDA, Syria except Sakha93 was obtained from Agric. Res. Cent., Egypt. The treatments consisted of two bacterial strains *Pseudomonas fluorescens* NBRC 14160 (B1) and *Bacillus megaterium* NBRC 15308 (B2) under two levels of saline solutions 4.69 ds m^−1^ or 3000 ppm and 7.81 ds m^−1^ or 5000 ppm were prepared with mixing NaCl and KCl and the osmotic potential of the solutions were determined with a conductivity meter. Tap water served as a control. Wheat seeds were surface sterilized by 2% sodium hypochlorite solution for 6 min then washed with 3 times by deionized sterilized water. The sterilized seeds were treated with a prepared bacterial suspension of *Pseudomonas fluorescens* NBRC 14160 (B1) and *Bacillus megaterium* NBRC 15308 (B2) for 3h. Seeds for each variety were sown in plastic pots (40 cm diameter and 50cm depth) and each pot was filled with a mixture of sand and farm yard manure in the proportion of 2:1 by volume which was mixed well with bacterial culture (10^8^ CFU ml^−1^). The chemical characters of soil are presented in Table 1 and temperature range and relative humidity range (Figure 1) by [26]. Each pot was planted with 20 seeds and pots were irrigated once every 5 days in average with saline solution or tap water (control). To estimate salinity concentrations with bacterial strains effects on seed germination and seedling growth of six bread wheat cultivars a randomized complete block design was used with a factorial arrangement of treatments (cultivars and salinity levels with bacterial strains) with four replicates.

### 2.7. The following characters were studied

Germination percentage, the data on germination was taken at 10 and 15 days after sowing and the relative percentage of germination was calculated from the following equation number of germinated Seeds / Total number of seeds ×00. After 60 days the shoot length (cm), root length (cm) were recorded and the shoot and root dry weights of five plants were taken after drying the samples from each genotype in each treatment in electric oven for 72 h at 70 °C. Root / shoot ratio: This ratio was calculated for weights by dividing root values by shoots. Chlorophyll SPAD value: SPAD values (The SPAD-502 chlorophyll meter) (Minolta Co., Ltd., Japan), a portable, self-calibrating, convenient, and nondestructive device which can be used for measuring the amount of chlorophyll present in plant leaves at the flowering stage [27]. Peroxidase (POD) activity was assayed by taking samples from leaves (0.5 g) of five plants in each treatment for each variety. POD activity was determined as described by [28]. One unit of POD activity was defined as an absorbance change of mg fresh weight per min. Changes in absorbance of the reaction solution were determined at 470 nm every 30 sec. K+ and Na+ (mg/g) were measured using standard flame photometer procedure [29]. The ratio K+/Na+ was calculated.

**Table 1.** Chemical properties of the experimental soil.

CaCO_3_ %	pH	Cations meq/L				Anions meq/L		
		Ca^+^	Mg^+^	Na^+^	K^+^	Cl^−^	So_4_^2−^	HCO_3_^−^
0.52	7.15	5.1	3.6	19.4	0.70	15	8.1	2.1

### 2.8. Statistical analysis

Data were arranged and statically observed through two-ways of analysis of variance (ANOVA) [30]. Treatments mean variation under different salinity treatments were observed by using least significant difference (LSD) at the 0.05 level of significance.

### 2.9. Phenotype and Genetic Variability parameters

Genotypic and phenotypic coefficient of variability, heritability in broad sense and genetic advance (GA) were determined by the formula given by [31]. Computer program software TNAUSTAT-Statistical package was used for estimating genetic parameters [32].

$$\text{Phenotypic coefficients of variation: PCV}=\frac{\sqrt{\sigma^{2}\text{px}}}{\text{x}}x100$$

$$\text{Genotypic coefficients of variation: GCV}=\frac{\sqrt{\sigma^{2}\text{gx}}}{\text{x}}x100$$

where,

σ2p = phenotypic variance

σ2g = genotypic variance

x = grand mean of a character.

Estimation of heritability in broad sense: Broad sense heritability (h 2) expressed as the percentage 154 of the ratio of the genotypic variance (σ2 g) to the phenotypic variance (σ2p) and was estimated on 155 genotype mean basis as described by[33] as:

$${}^{\text{h2}}\text{B}{=}^{\sigma \text{2}}\text{g}{/}^{\sigma \text{2}}\text{px1}00$$

where,

^h 2^ B = Heritability in Broad sense

σ2p = phenotypic variance.

σ2g = genotypic variance.

Estimation of genetic advance: Genetic advance (GA) and percent of the mean (GAM), assuming selection of superior 5% of the genotypes was estimated in accordance with the methods illustrated 164 by [34] as:

where,

$$\text{GA}=\frac{K*\sqrt{\sigma^{2}}\text{p*σ}2g}{\sigma 2\;p}$$

GA = expected genetic advance

k = the standardized selection differential at 5% selection intensity (K = 2.063)

σ2p = phenotypic variance

σ2g = genotypic variance.

## 3. Results and discussion

### 3.1. Salt tolerance assay of bacteria

The four strains were tested *in vitro* for abiotic stress at elevated salt concentration (1–10%). The results revealed that the four strains could be able to tolerate salinity levels up to 3% NaCl while the strains *Pseudomonas fluorescens* NBRC 14160 and *Bacillus megaterium* NBRC 15308 grow on NA media supplemented with 6% NaCl.

### 3.2. The evaluated strains produced Ammonia, IAA, and ACC

**Figure 2.**
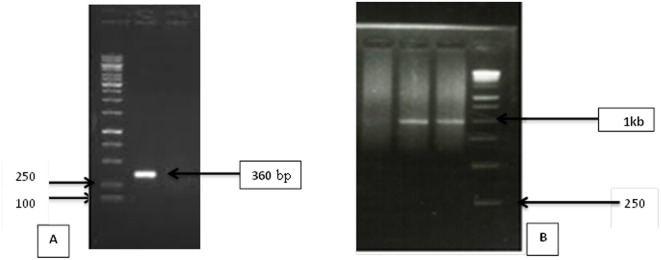
*Pseudomonas fluorescens* NBRC 14160 positive for *nifH* gene amplification (A) while *Pseudomonas fluorescens* NBRC 14160 and *Bacillus megaterium NBRC 15308* strains showed the amplification of acdS gene fragment (B) in Agarose gel photograph

These selected strains were screened *in vitro* to their ability to produce IAA, nitrogen fixation, and ACC deaminase activity. The results of four strains showed different PGP traits. All tested strains were able to synthesize IAA in the absence of L-tryptophan. Out of the 4 strains, *Pseudomonas fluorescens* NBRC 14160 and *Bacillus megaterium* NBRC 15308 produced ACC deaminase. Only strain *Pseudomonas fluorescens* NBRC14160 could produce ammonia as evidenced by clear blue coloration. Furthermore, it has *nifH* gene amplification, producing an amplified fragment of about 360 bp (Figure 2). Amplifications of *acds* gene was observed (1kb) in both strains (*Pseudomonas fluorescens* NBRC 14160 and *Bacillus megaterium* NBRC 15308) which confirmed the presence of *acds* gene (Figure 2).Results disagree with research, which confirmed the inability of *Pseudomonas* spp. to fix nitrogen [35]–[37]. However, Results agree with research has confirmed that *Pseudomonas fluorescens* NBRC 14160 have the capability to fix nitrogen. Pseudomonas have been widely reported as NFB of nonlegumes [38],[39]. Previous studies have shown that plant growth promoting bacteria are able to not only fix nitrogen but also produce IAA and ACC deaminase [40],[41].

### 3.3. Swimming and swarming activity

Bacterial swimming and swarming motility has been shown quantitatively. After 48h incubation, the four strains showed positivity for swimming motility. *Pseudomonas fluorescens* NBRC 14160 showed the greatest swimming motility 58 mm while *Serratia liquefaciens* ATCC 27592 showed the least swimming motility 33 mm. All the strains showed positivity for swarming motility except *Serratia liquefaciens* ATCC 27592. *Bacillus megaterium* NBRC 15308 showed the least swarming motility with swarm diameter up to 23 mm. On the other hand, *Pseudomonas fluorescens* NBRC 14160 exhibited greater swarming motility 48 mm as compared with the other strains **(**Table 2).

**Table 2.** Results of swimming and swarming motilities of bacterial strains.

Bacterial strains	Swimming motility	Swarming motility
*Pseudomonas fluorescens* NBRC 14160	58	48
*Serratia liquefaciens ATCC 27592*	33	-
*Bacillus subtilis SBMP4*	55	42
*Bacillus megaterium NBRC 15308*	49	23

### 3.4. Analysis of variance

Analysis of variance was carried out for all morphological and physiological characters as illustrated in Table 3. Analysis of variance showed significant differences between treatments (salinity levels and bacterial strains), genotypes and their interaction between genotypes × treatments for all the characters except shoot dry weight, root dry weight and root/shoot ratio at significant level p < 0.05.

### 3.5. Mean performance

Mean performance for morphological and physiological characters for all genotypes under salinity and bacterial strain are given in Tables 3–5 and 6. All characters of the studied genotypes were affected by salinity stress; increasing salinity levels caused reduction in the mean performance for all traits except the activity of peroxidase enzyme (POD).

### 3.6. Germination percent

Germination percentage trait is considered the first indicator for screening and differentiates between tolerance and sensitive genotype. Studying wheat genotypes showed difference responses for control, 3000 ppm and 5000 ppm treatments. Results showed that high levels of salinity caused decreasing in germination percent reach to 30% at 5000 ppm. While Sakha93 and line 432 have the highest mean values under all saline and non-saline conditions. These results accepted with [42] revealed to gradual reduction in germination percent and inhibition of seedling growth in studied cultivars of wheat related with increasing of salinity concentrations. The bacterial strains B1 and B2 treatments gave increasing in mean values for all genotypes compared with their corresponding and Sakha93 had the maximum value (89.56) followed by line 432 (87.47) under normal and salinity stress conditions (Table 4). Fluorescent pseudomonad strains that possess ACC deaminase activity have the selective advantage over other bacteria during biotic [43] and abiotic stress conditions [44],[45] which have positive effect on embryo germination.

### 3.7. Shoot and root length

Mean values of shoot and root length are presented in Table (4) indicated that shoot length (SL) of various genotypes under different treatments ranged from 24.53 (cm) for line 404 to 27.35 (cm) for Sakha 93 genotype. Increasing salinity levels decreased shoot length significantly than control; plant uses this shorting mechanism to keep the highest amount of water in its tissues and decreasing the transpiration process. However, the genotypes were treated with the bacterial strains B1 and B2 gave the highest values for shoot length compared with their corresponding under different salinity levels. According to root length, early and rapid elongation of root is important indication of salinity tolerance. In the present investigation, the root length also significantly declined with increased external salinity concentration (Table 4) and consequently, all treatments caused a decreasing in root elongation in all genotypes compared to their controls. These results agreement with [46], [47]. The genotypes with the bacterial strains B1 and B2 gave the highest values for root length compared with their corresponding under different salinity levels. In all the stress conditions the mean root length for all genotypes varied from 12.72 cm (line 404) to 14.10 cm (Sakha93). Previous studies have shown the ability of PGPR had to generate IAA has been related to improve wheat growth under saline conditions [48],[49] and it is similar to previous data was recorded by several workers who confirmed that inoculation with PGPR (Pseudomonas and Bacillus) to wheat improved plant production, yield, and physiological attributes [50],[51]. Our results agreement with [52],[53] who found that Bacteria containing ACC deaminase activity would act to reduce the level of stress ethylene and thus could resistance to various stresses and insignificant improvement in plant growth characters was observed in Ps. Fluorescens strain TDK1 possessing ACC deaminase treatment against saline stress. The use of PGPR with nitrogen fixing capability is considered a good strategy for boosting salt-sensitive plant growth [54].

### 3.8. Shoot and root dry weight

Salinity has negative effect on weight of plants, which cause many difficult of water and nutrients absorption from soil subsequently delayed in growth and decreasing in rate of cell divisions [55]. Some studied genotypes showed high effect with sever salinity level reached to 60% like line 404 and line 356 for root dry weight while Sakha93 and line 380 have slight effect with sever condition (Table 4). B1 and B2 caused reduction of salinity effects and increase dry weight for some studied lines of wheat this increasing reach to 20% over plants of control condition in Sakha93 and 10–15% in other studied lines (Table 5). Using of plant growth-promoting fluorescent pseudomonads which decrease the damage to plants that occurs under saline stress conditions is a potentially important adjuvant to agricultural practice in locales where salinity is a major constraint [56].

**Table 3.** Analysis of variance for the studied traits in six wheat genotypes in response to water salinity stress.

Source	D.F	Ger.%	Shoot length	Root length	Shoot dry	Root dry weight	Root/shoot ratio	Chlorophyll	POD activity	Na	K	K/Na
Varieties (V)	5	**	**	**	Ns	Ns	Ns	**	**	**	**	**
Treatments(T)	8	**	**	**	Ns	Ns	**	**	**	**	**	**
V×T	40	**	*	Ns	Ns	Ns	Ns	**	**	**	**	**

NS = not significant *=significant **= highly significant

**Table 4.** Effect of inoculation with two bacterial strains, *Pseudomonas fluorescens NBRC 14160* and *Bacillus megaterium NBRC 15308*, on germination%, shoot and root length (cm) in six wheat genotypes under different salinity levels.

Germination%
		Line 404	Line 356	Line 420	Line 432	Sakha 93	Line 380	Mean
Con	Control	93.87 ± 1.73	90.73 ± 1.2	90.73 ± 1.2	98.00 ± 2.1	96.00 ± 1.2	90.73 ± 1.2	93.34
	B1	95.00 ± 0.75	98.04 ± 0.75	98.04 ± 0.75	96.56 ± 1.84	98.04 ± 0.75	98.04 ± 1.75	97.29
	B2	98.04 ± 1.8	93.87 ± 0.99	93.87 ± 0.86	96.81 ± 2.06	96.00 ± 0.89	93.87 ± 1.8	95.41
3000	Control	84.48 ± 0.5	84.48 ± 0.60	84.48 ± 0.45	84.48 ± 0.63	87.26 ± 0.98	84.48 ± 1.80	84.94
	B1	90.37 ± 1.14	90.37 ± 1.14	90.37 ± 1.14	96.00 ± 1.73	90.73 ± 1.14	90.37 ± 1.14	91.37
	B2	87.26 ± 0.68	87.26 ± 0.68	87.26 ± 0.68	87.26 ± 0.68	90.37 ± 0.68	87.26 ± 1.68	87.78
5000	Control	65.00 ± 1.6	65.00 ± 0.67	76.22 ± 1.7	65.00 ± 1.4	81.67 ± 1.4	65.00 ± 1.9	69.65
	B1	81.48 ± 2.56	81.48 ± 2.56	77.03 ± 1.93	81.48 ± 2.56	84.48 ± 2.56	81.48 ± 2.565	81.24
	B2	81.67 ± 1.53	81.67 ± 1.53	68.69 ± 1.28	81.67 ± 1.53	81.48 ± 1.53	81.67 ± 1.5	79.48
Mean		86.35	85.88	85.19	87.47	89.56	85.88
L.S.D 0.05		V= 1.13 T= 2.16 V×T=340			
Short length	
Con	Control	28.70 ± 1	27.83 ± 0.58	28.83 ± 1.7	28.50 ± 1.4	30.50 ± 1.47	28.43 ± 1.15	28.80
	B1	29.34 ± 1.53	28.83 ± 1.12	29.50 ± 1.53	29.17 ± 2.08	33.83 ± 1.56	30.17 ± 1.73	30.14
	B2	28.17 ± 1.4	28.50 ± 1.53	28.83 ± 3.36	28.50 ± 1.53	31.34 ± 1.76	28.86 ± 1.53	29.03
3000	Control	24.50 ± 3.06	22.23 ± 2.65	23.83 ± 0.58	22.50 ± 0.56	26.83 ± 1.12	23.50 ± 3.2	23.90
	B1	27.50 ± 2.28	27.50 ± 3.51	27.88 ± 0.84	27.83 ± 1.3	29.33 ± 1.56	28.10 ± 1.58	28.02
	B2	26.83 ± 2.16	27.17 ± 2.8	26.90 ± 1.53	27.83 ± 1.56	28.17 ± 2	27.70 ± 1.78	27.43
5000	Control	14.07 ± 2	16.17 ± 1.48	15.83 ± 1.2	16.83 ± 2	18.17 ± 1.24	17.50 ± 1.6	16.43
	B1	21.50 ± 2.4	22.83 ± 1.53	21.51 ± 0.98	22.90 ± 1.5	24.50 ± 1.52	21.83 ± 2.01	22.51
	B2	20.17 ± 0.91	22.20 ± 0.63	20.39 ± 1.5	22.50 ± 2.08	23.50 ± 1.57	20.77 ± 2.77	21.59
Mean		24.53	24.81	24.83	25.17	27.35	25.21
L.S.D 0.05		V= 1.12 T= 1.15 V×T=1.39			
Root length	
Con	Control	14.67 ± 1.4	15.33 ± 0.68	15.60 ± 1.18	16.30 ± 1.2	16.33 ± 1.6	15.00 ± 1.15	15.54
	B1	16.60 ± 1.72	16.30 ± 1.1	17.10 ± 1.70	17.00 ± 1.53	17.67 ± 1.14	17.33 ± 1.73	17.00
	B2	15.50 ± 1.56	16.00 ± 0.88	16.40 ± 2.36	16.50 ± 1.50	16.00 ± 1.21	15.33 ± 1.53	15.96
3000	Control	11.33 ± 3.06	11.20 ± 2.65	12.30 ± 0.81	12.00 ± 1.5	13.50 ± 1	13.00 ± 3.21	12.22
	B1	14.20 ± 2.16	14.20 ± 3	15.00 ± 0.84	15.37 ± 1.15	15.50 ± 0.58	14.50 ± 1.53	14.80
	B2	13.60 ± 2.28	13.90 ± 2.52	14.80 ± 1.53	15.20 ± 2	15.00 ± 2	13.70 ± 1.80	14.37
5000	Control	7.60 ± 2	8.33 ± 1.53	8.80 ± 1.20	8.45 ± 1.3	9.60 ± 1.4	9.20 ± 1.58	8.66
	B1	10.67 ± 2.1	10.90 ± 1.53	11.80 ± 1.35	11.40 ± 1.53	11.67 ± 1.58	11.33 ± 2.77	11.30
	B2	10.33 ± 1.9	10.50 ± 3.06	10.89 ± 1.8	11.00 ± 1.53	11.60 ± 2.65	11.10 ± 2	10.90
Mean		12.72	12.96	13.63	13.69	14.10	13.39
L.S.D 0.05		V= 1.14 T= 1.17 V×T=2.43			

**Table 5.** Effect of inoculation with two bacterial strains, *Pseudomonas fluorescens NBRC 14160* and *Bacillus megaterium NBRC 15308*, on Shoot dry weight (g), Root Dry weight (g) and Root/ shoot Dry weight in six wheat genotypes under different salinity levels.

Shoot dry weight	
		Line 404	Line 356	Line 420	Line 432	Sakha	Line 380	mean
Con	Control	0.213 ± 0.01	0.200 ± 0.16	0.212 ± 0.20	0.200 ± 0.01	0.221 ± 0.02	0.210 ± 0.01	0.209
	B1	0.225 ± 0.02	0.214 ± 0.20	0.220 ± 0.24	0.210 ± 0.01	0.270 ± 0.02	0.230 ± 0.02	0.228
	B2	0.217 ± 0.01	0.210 ± 0.17	0.250 ± 0.20	0.210 ± 0.02	0.249 ± 0.01	0.223 ± 0.02	0.227
3000	Control	0.155 ± 0.22	0.147 ± 0.21	0.150 ± 0.22	0.140 ± 0.01	0.199 ± 0.01	0.174 ± 0.04	0.161
	B1	0.213 ± 0.20	0.189 ± 0.19	0.200 ± 0.14	0.200 ± 0.01	0.210 ± 0.01	0.200 ± 0.02	0.202
	B2	0.200 ± 0.19	0.180 ± 0.20	0.182 ± 0.18	0.180 ± 0.01	0.191 ± 0.02	0.188 ± 0.01	0.187
5000	Control	0.112 ± 0.21	0.108 ± 0.24	0.111 ± 0.14	0.106 ± 0.02	0.137 ± 0.02	0.120 ± 0.01	0.116
	B1	0.19 ± 0.20	0.144 ± 0.17	0.146 ± 0.19	0.134 ± 0.01	0.190 ± 0.01	0.170 ± 0.03	0.156
	B2	0.147 ± 0.20	0.141 ± 0.16	0.144 ± 0.15	0.133 ± 0.02	0.177 ± 0.03	0.169 ± 0.02	0.152
Mean		0.181	0.17	0.179	0.168	0.205	0.187
L.S.D 0.05		V= 0.015 T=0.019 V×T=0.046				
Root Dry weight	
Con	Control	0.145 ± 0.01	0.140 ± 0.01	0.148 ± 0.04	0.137 ± 0.01	0.162 ± 0.01	0.156 ± 0.01	0.148
	B1	0.156 ± 0.02	0.131 ± 0.01	0.159 ± 0.01	0.150 ± 0.01	0.190 ± 0.01	0.166 ± 0.02	0.159
	B2	0.150 ± 0.01	0.129 ± 0.01	0.150 ± 0.01	0.143 ± 0.01	0.171 ± 0.01	0.159 ± 0.01	0.150
3000	Control	0.106 ± 0.03	0.092 ± 0.02	0.110 ± 0.01	0.100 ± 0.01	0.130 ± 0.01	0.116 ± 0.01	0.109
	B1	0.141 ± 0.01	0.136 ± 0.03	0.143 ± 0.01	0.134 ± 0.01	0.157 ± 0.01	0.151 ± 0.01	0.144
	B2	0.137 ± 0.01	0.134 ± 0.02	0.136 ± 0.01	0.133 ± 0.02	0.148 ± 0.02	0.142 ± 0.01	0.138
5000	Control	0.072 ± 0.02	0.065 ± 0.01	0.080 ± 0.01	0.074 ± 0.01	0.091 ± 0.01	0.081 ± 0.01	0.077
	B1	0.104 ± 0.02	0.078 ± 0.01	0.109 ± 0.01	0.100 ± 0.01	0.116 ± 0.01	0.100 ± 0.03	0.101
	B2	0.101 ± 0.01	0.074 ± 0.03	0.102 ± 0.01	0.090 ± 0.01	0.110 ± 0.02	0.091 ± 0.02	0.095
Mean		0.124	0.109	0.126	0.118	0.142	0.129
L.S.D 0.05		V= 0.03 T= 0.04 V×T=0.09				
Con	Control	0.68 ± 0.02	0.63 ± 0.01	0.70 ± 0.01	0.76 ± 0.01	0.71 ± 0.02	0.68 ± 0.02	0.70
	B1	0.67 ± 0.01	0.65 ± 0.01	0.71 ± 0.02	0.57 ± 0.02	0.33 ± 0.02	0.67 ± 0.02	0.60
	B2	0.68 ± 0.01	0.63 ± 0.01	0.68 ± 0.04	0.67 ± 0.02	0.70 ± 0.01	0.67 ± 0.01	0.67
3000	Control	0.59 ± 0.02	0.71 ± 0.03	0.68 ± 0.01	0.92 ± 0.01	0.71 ± 0.01	0.65 ± 0.03	0.71
	B1	0.62 ± 0.01	0.70 ± 0.03	0.64 ± 0.01	0.50 ± 0.01	0.70 ± 0.01	0.65 ± 0.01	0.63
	B2	0.68 ± 0.01	0.63 ± 0.03	0.67 ± 0.02	0.60 ± 0.01	0.67 ± 0.02	0.70 ± 0.01	0.66
5000	Control	0.71 ± 0.02	0.71 ± 0.01	0.57 ± 0.02	0.33 ± 0.01	0.68 ± 0.02	0.60 ± 0.01	0.60
	B1	0.65 ± 0.01	0.68 ± 0.01	0.68 ± 0.01	0.63 ± 0.01	0.70 ± 0.01	0.67 ± 0.03	0.67
	B2	0.70 ± 0.01	0.70 ± 0.03	0.60 ± 0.01	0.64 ± 0.02	0.69 ± 0.03	0.67 ± 0.02	0.67
Mean		0.67	0.67	0.66	0.63	0.66	0.66
L.S.D 0.05		V = 0.75 T = 0.92 V×T = 2.244				

### 3.9. Chlorophyll content

Chlorophyll is one of the most important chloroplast components for photosynthesis, because it harvests the light and produces reducing powers; however, Chlorophyll is susceptible to salt stress and may affect plant yield and quality. The previous research found a significant positive relationship between SPAD values and the grain yield under salinity stress [57],[58]. Results showed sever reduction in chlorophyll content for all studied genotypes of wheat under salinity stress (Table 6). Sakha93 showed the lowest reduction followed by line 404 and line 420. The inhibitory effects of salt stress on chlorophyll pigments could be due to suppression of specific enzymes responsible for the synthesis of the green pigments, or due to increased chlorophyllase activity in wheat mustard respectably [59]–[61]. In this research B1 and B2 treatments showed induction effect by enhancement enzymes of photosynthesis in plants of wheat. Tolerant genotypes had minimum reduction in chlorophyll content as compared to susceptible cultivars and the cumulative effect of these changes lead to amelioration of salinity stress tolerance in them [62]. High salinity decreased net photosynthetic rate, transpiration rate, and stomatal conductance whereas salt-sensitive cultivars had the lowest net photosynthetic rate, stomatal conductance [63].

**Table 6.** Effect of inoculation with two bacterial strains, *Pseudomonas fluorescens NBRC 14160* and *Bacillus megaterium NBRC 15308*, on Chlorophyll and POD activity in six wheat genotypes under different salinity levels.

Chlorophyll	
		Line 404	Line 356	Line 420	Line 432	Sakha	Line 380	Mean
Con	Control	61.97 ± 5.65	57.6 ± 3.27	62.23 ± 2.47	60.69 ± 2.15	65.1 ± 4.77	60.98 ± 5.06	61.43
	B1	63.57 ± 5.44	58.3 ± 4.50	63.52 ± 2.03	63.5 ± 3	70.1 ± 3.20	64.27 ± 4.84	63.88
	B2	61.63 ± 2.49	57.83 ± 2.05	63.18 ± 4.64	62.23 ± 3.25	67.23 ± 3.48	61.01 ± 3.86	62.19
3000	Control	53.83 ± 2.86	50.43 ± 6.39	51.22 ± 3.77	50.21 ± 3.12	54 ± 1.52	49.63 ± 2.39	51.55
	B1	59.5 ± 2.02	55.57 ± 3.58	59.23 ± 2.92	58.73 ± 3.89	63.87 ± 2.78	59.39 ± 2.75	59.38
	B2	59.1 ± 1.76	54.95 ± 5.44	57.22 ± 3.17	57.7 ± 4.20	61.67 ± 4.07	55.7 ± 2.14	57.72
5000	Control	44.5 ± 1.5	36.72 ± 4.70	39.1 ± 1.38	37.74 ± 1.98	46.5 ± 3.47	39.73 ± 2.05	40.72
	B1	55.37 ± 5.83	49.76 ± 1.22	50.55 ± 1.71	51 ± 3.50	52.63 ± 2.75	49.5 ± 3.55	51.47
	B2	55.3 ± 4.90	47.06 ± 2.02	49.64 ± 1.41	48.86 ± 1.50	56.7 ± 2.67	49.49 ± 4.77	51.18
Mean		57.2	52.02	55.1	54.52	59.76	54.41
L.S.D 0.05		V = 1.13 T = 2.16 V×T=2.42				
POD activity	
Con	Control	45.41 ± 0.33	32.98 ± 0.42	35.95 ± 0.52	46.43 ± 1.74	44.14 ± 0.51	44.96 ± 1.99	41.64
	B1	36.33 ± 0.15	24.79 ± 0.88	32.81 ± 0.64	32.72 ± 1.25	32.81 ± 0.25	45.06 ± 1.80	34.08
	B2	40.38 ± 1.03	32.41 ± 0.90	34.49 ± 0.84	39.13 ± 2.86	42.96 ± 0.84	49.68 ± 1.39	39.84
3000	Control	56.07 ± 1.93	40.82 ± 0.11	61.45 ± 0.57	57.81 ± 1.26	53.83 ± 0.13	60.26 ± 0.75	55.04
	B1	50.05 ± 1.63	34.47 ± 0.70	41.29 ± 1.54	52.25 ± 0.84	51.02 ± 0.77	51.19 ± 0.62	46.71
	B2	52.9 ± 1.16	36.92 ± 0.81	54.87 ± 1.08	53.36 ± 0.64	52.88 ± 0.18	53.95 ± 0.93	50.81
5000	Control	64.36 ± 1.83	53.82 ± 1.64	73.06 ± 0.74	67.77 ± 1.86	69.49 ± 0.38	73.06 ± 0.74	66.93
	B1	60.69 ± 0.93	44.78 ± 1.14	62.38 ± 0.58	62.14 ± 1.05	62.59 ± 0.40	64.61 ± 1.02	59.53
	B2	62.07 ± 1.03	48.5 ± 1.85	68.58 ± 1.5	63.89 ± 1.28	64.78 ± 0.64	68.58 ± 1.5	62.73
Mean		52.03	38.83	51.65	52.83	52.72	56.81
L.S.D 0.05		V = 1.23 T = 1.50 V×T = 3.45					

**Table 7.** Effect of inoculation with two bacterial strains, *Pseudomonas fluorescens NBRC 14160* and *Bacillus megaterium NBRC 15308*, on Na (mg g^−1^ DW), K (mg g^−1^ DW) and K/Na in six wheat genotypes under different salinity levels.

Na (mg g^−1^ DW)								
		Line 404	Line 356	Line 420	Line 432	Sakha	Line 380	Mean
Con	Control	47.57 ± 1.23	46.51 ± 1.36	46.45 ± 5.41	46.01 ± 1.75	49.17 ± 1.79	49.38 ± 3.14	47.52
	B1	40.12 ± 2.12	43.73 ± 2.50	43.50 ± 5.18	43.30 ± 2.14	42.12 ± 1.95	41.12 ± 2.26	42.32
	B2	41.30 ± 1.27	44.93 ± 1.93	44.99 ± 6	45.18 ± 1.30	45.47 ± 1.51	43.61 ± 2.58	44.25
3000	Control	66.77 ± 5.20	65.00 ± 1.5	69.52 ± 5.69	65.43 ± 1.52	65.17 ± 1.24	70.92 ± 3.46	67.14
	B1	57.87 ± 4.01	58.23 ± 2.90	63.04 ± 5.22	56.30 ± 2.54	63.13 ± 1.43	61.65 ± 4.73	60.04
	B2	64.00 ± 5.49	63.24 ± 3.59	63.90 ± 4.27	58.23 ± 1.35	63.93 ± 1.69	70.35 ± 3.31	63.94
5000	Control	77.20 ± 4.06	74.38 ± 3.14	77.90 ± 1.22	74.47 ± 1.80	72.13 ± 1.77	80.46 ± 5.33	76.09
	B1	73.43 ± 3.50	72.34 ± 3.50	73.12 ± 1.92	69.93 ± 1.32	70.94 ± 1.32	76.73 ± 5.26	72.75
	B2	74.13 ± 1.27	73.38 ± 3.14	76.26 ± 1.86	70.27 ± 1.68	71.27 ± 1.59	76.82 ± 5.01	73.69
Mean		60.27	60.19	62.08	58.79	60.37	63.45	
L.S.D 0.05		V = 0.13 T = 0.16 V × T = 1.40						
K (mg g^−1^ DW)								
Con	Control	40.60 ± 0.71	40.37 ± 1.15	43.36 ± 3.52	42.63 ± 2.24	46.63 ± 2.11	47.10 ± 2.20	43.45
	B1	40.57 ± 0.40	39.78 ± 1.5	41.90 ± 2.27	38.80 ± 3.75	39.70 ± 1.27	39.75 ± 2.04	40.08
	B2	39.84 ± 0.81	41.42 ± 1.84	41.16 ± 2.39	42.23 ± 1.94	42.84 ± 1.80	42.14 ± 2.37	41.60
3000	Control	56.00 ± 1.49	54.46 ± 1.63	44.80 ± 2.99	33.20 ± 1.15	57.79 ± 1.31	48.73 ± 1.46	49.16
	B1	46.73 ± 1.25	51.96 ± 1.46	41.17 ± 1.44	40.37 ± 1.46	60.85 ± 3.30	48.03 ± 1.17	48.19
	B2	53.39 ± 1.10	53.62 ± 1.64	43.50 ± 2.84	40.00 ± 1.53	54.27 ± 1.64	47.95 ± 2.30	48.79
5000	Control	47.27 ± 0.32	57.46 ± 2.89	41.50 ± 1.05	44.30 ± 1.57	62.05 ± 1.37	49.49 ± 2.31	50.35
	B1	53.91 ± 0.90	53.43 ± 1.78	42.40 ± 1.11	41.44 ± 1.54	57.24 ± 1.66	48.52 ± 3.29	49.49
	B2	40.90 ± 0.95	55.01 ± 2.27	52.28 ± 1.68	43.77 ± 1.57	61.06 ± 1.68	49.38 ± 2.35	50.40
Mean		46.58	49.72	43.56	40.75	53.60	46.79	
L.S.D 0.05		V = 2.12 T = 2.15 V × T = 1.38						
Con	Control	0.850 ± 0.97	0.870 ± 0.76	0.930 ± 2.46	0.930 ± 3.5	0.950 ± 1.95	0.950 ± 2.17	0.910
	B1	1.010 ± 2.76	0.910 ± 1.5	0.960 ± 2.22	0.900 ± 2.95	0.940 ± 1.11	0.970 ± 3.15	0.950
	B2	0.960 ± 1.04	0.920 ± 1.39	0.910 ± 2.69	0.930 ± 1.62	0.940 ± 0.66	0.970 ± 2.47	0.940
3000	Control	0.840 ± 3.25	0.840 ± 1.06	0.640 ± 2.34	0.510 ± 0.84	0.890 ± 0.77	0.690 ± 2.96	0.730
	B1	0.730 ± 3.62	0.820 ± 1.2	0.640 ± 2.33	0.690 ± 0.44	0.950 ± 2.37	0.680 ± 2.95	0.750
	B2	0.920 ± 3.29	0.920 ± 2.12	0.690 ± 2.55	0.710 ± 0.50	0.860 ± 1.17	0.780 ± 2.80	0.810
5000	Control	0.610 ± 2.19	0.770 ± 1.02	0.530 ± 1.13	0.590 ± 1.19	0.860 ± 1.57	0.620 ± 2.32	0.660
	B1	0.730 ± 2.20	0.740 ± 2.69	0.580 ± 1.02	0.590 ± 1.43	0.810 ± 1.49	0.630 ± 2.28	0.680
	B2	0.550 ± 1.11	0.750 ± 2.71	0.690 ± 1.77	0.620 ± 1.13	0.860 ± 1.13	0.640 ± 2.68	0.680
Mean		0.800	0.840	0.730	0.720	0.900	0.770	
L.S.D 0.05		V = 0.02 T = 0.03 V × T = 0.07						

### 3.10. POD activity

Peroxidase activity (POD) enzyme is an important physiological repair mechanisms or process in living cells, which eliminate or scavenging the reactive oxygen species (ROS). The sources of these ROS from secondary metabolism process and from some types of stress like salinity. ROS destroy the vital component in living cell like proteins membranes and DNA [64],[65]. Results showed (Table 6) increasing in POD activity values by increasing the levels of salinity and the response of lines are differed; line 380 has high mean value while the lowest mean value showed in line 356. Indicated that line 380 have more ability to salinity tolerance. This finding agree with [66]–[68] who found increasing in activity of peroxidase under salinity treatments in tolerant cultivars. Using B1 and B2 treatments caused decreasing in POD activity especially line 356.

### 3.11. Sodium and Potassium content:

These elements are very important for plant life and production. Na element activates several metabolic processes in plant and the excess amount can cause toxicity in plant cell. The tolerant genotype has low content of Na; Sakha93 cultivars and line 432 showed the lowest mean values of Na content under all treatments. B1 and B2 treatments caused reduction in mean values of Na content comparing with control plants (Table 7). Sakha93 and line 356 showed increasing of potassium content with increasing the levels of salinity and they have the high mean value over other studied lines which are considered more tolerant plants (Table 7). Using B1 and B2 treatments caused decreasing in potassium content under saline and non-saline conditions (Table 7). Potassium element considered transporter in plant cell and activator for many enzymes. The deficiency of this element caused plant day and considerable reduction in quality and quantity of yield. K/Na ratio trait; Sakha93 showed high percent under most treatments followed by Line 356 while Line 432 and line 420 were showed the lowest values. The using B1 and B2 treatments caused increasing in K/Na ratio. These results agree with [47] showed that concentration of Na+ was increased with increasing concentration of salinity and mineral ion concentration including K+ in the leaves gradually decreased by increasing salinity levels to reach their lowest values at the greatest level of salinity. The inoculation by *B*. subtilis strain GB03 decreased gradually Na accumulation in bread wheat with improved K /Na ratio [69].

### 3.12. Genetic parameters

Some genetic parameters are estimated in this study; phenotypic and genotypic coefficient of variability, heritability in broad sense and genetic advance; PCV, GCV, h^2^ and GA respectively for some growth and physiological traits of wheat were presented in Table 8. Results showed that values of PCV were higher than GCV values for studied traits illustrate the role of environmental factors on these traits. Salinity stress was compared with normal condition which showed increasing in values of heritability. Germination percent, shoot length and potassium content traits had high values of heritability and genetic advance so these traits might use in selection of plant breeding programs for salinity tolerance. These Finding accepted with [70] in wheat under saline condition. Bacterial treatments comparing with normal condition showed low values of genetic parameters such as germination percent, root length and potassium content; but when it accompanied with salinity treatment caused increasing in values of genetic parameters for most traits.

**Table 8.** Genetic parameters: phenotypic and genotypic coefficient of variability (PCV and GCV), heritability in broad sense (h^2^) and genetic advance (GA) for growth and physiological traits under normal and stress conditions.

Traits	Genetic parameters	Con	Con + B1	Con + B2	Stress con	Stress + B1	Stress + B2
Ger%	PCV	3.4	1.35	1.93	10.61	2.95	6.58
	GCV	3.36	1.26	1.88	10.61	2.95	6.56
	h^2^	98.17	87.27	94.59	98.97	99.84	97.57
	GA	6.41	2.37	3.59	15.27	4.95	10.73
Shoot length	PCV	3.44	6.35	4.09	8.39	6.09	6.37
	GCV	2.99	6.14	4	8.35	5.58	6.35
	h^2^	75.52	93.42	95.6	96.19	90.91	93.61
	GA	1.54	3.69	2.34	2.85	0.03	2.86
Root length	PCV	4.84	3.62	3.72	7.45	35.12	3.97
	GCV	3.92	2.46	2.1	7.4	34.19	3.77
	h^2^	65.49	46.04	31.72	93.63	94.79	90.03
	GA	1.02	0.59	0.39	1.34	1.63	0.82
Chl. Spad	PCV	4.01	5.9	4.97	9.43	4.77	7.46
	GCV	3.95	5.86	4.92	9.42	3.78	7.45
	h^2^	96.74	98.6	97.88	97.84	63	99.82
	GA	4.91	7.66	6.24	7.95	3.19	7.89
Na content	PCV	3.27	3.95	3.59	3.61	2.52	3.49
	GCV	3.22	3.91	3.52	3.61	2.5	3.49
	h^2^	96.51	98.15	96.56	95.67	98.42	99.79
	GA	4.74	6.08	5.26	3.16	2.44	3.19
K content	PCV	6.74	2.75	2.71	18.54	13.11	12.3
	GCV	6.66	2.53	2.51	18.54	13.11	12.3
	h^2^	97.84	84.82	85.56	92.99	99.98	98.98
	GA	5.91	1.93	1.99	18.87	13.43	12.42

## Conclusion

The present study illustrates the Four PGPR strains possess some growth promoting .The selected strains were used to study their reflection *in vivo* on their ability to reduce the negative effects of high salinity on wheat plants growth. The production of ACC deaminase and other PGP traits by these strains improve plant growth under both non-saline and saline soils. In the future the selected PGPR strains could be used commercially to increase the productivity of wheat and other crops under saline conditions.
